# Clinical significance of epithelial mesenchymal transition (EMT) in chronic obstructive pulmonary disease (COPD): potential target for prevention of airway fibrosis and lung cancer

**DOI:** 10.1186/s40169-014-0033-2

**Published:** 2014-11-11

**Authors:** Sukhwinder Singh Sohal, Malik Quasir Mahmood, Eugene Haydn Walters

**Affiliations:** NHMRC Centre of Research Excellence for Chronic Respiratory Disease and Lung Ageing, School of Medicine, University of Tasmania, MS-1, 17 Liverpool Street Private Bag-23, Hobart, TAS 7000 Australia

**Keywords:** COPD, Lung cancer, Small airways, Large airways, Airway remodelling, Fibrosis, Epithelium, Rbm fragmentation, Smoking, Fibroblasts/myofibroblasts

## Abstract

Unfortunately, the research effort directed into chronic obstructive pulmonary disease (COPD) has been disproportionately weak compared to its social importance, and indeed it is the least researched of all common chronic conditions. Tobacco smoking is the major etiological factor. Only 25% of smokers will develop “classic” COPD; in these vulnerable individuals the progression of airways disease to symptomatic COPD occurs over two or more decades. We know surprisingly little about the pathobiology of COPD airway disease, though small airway fibrosis and obliteration are likely to be the main contributors to physiological airway dysfunction and these features occur earlier than any subsequent development of emphysema. One potential mechanism contributing to small airway fibrosis/obliteration and change in extracellular matrix (ECM) is epithelial mesenchymal transition (EMT), so called Type-II EMT. When associated with angiogenesis (Type-III EMT) it may well also be a link with the development of lung (airway) cancer which is closely associated with COPD. Active EMT in COPD may help to explain why lung cancer is so common in smokers and also the core pathophysiology of small airway fibrosis. Better understanding may lead to new markers for incipient neoplasia, and better preventive management of patients. There is serious need to understand key components of airway EMT in smokers and COPD, and to demarcate novel drug targets for the prevention of lung cancer and airway fibrosis, as well as better secondary management of COPD. Since over 90% of human cancer arises in epithelia and the involvement of EMT in all of these may be a central paradigm, insights gained in COPD may have important generalizable value.

## Background

Epithelial mesenchymal transition (EMT) is a process in which epithelial cells undergo a transition to a motile mesenchymal phenotype [1]. It can be considered as a marker of profound epithelial plasticity [2]. Elizabeth Hay from Harvard University was the first to describe the process of “epithelial mesenchymal transformation” in 1982, and then in 1995 as an important process involved in embryogenesis and organ development [3-5]. In the intervening time, the term “transformation” had been replaced with the term “transition”, indicating potential induction and reversibility of the process, even potentially a two-way process from mesenchymal cells to epithelium [6]. This reverse process has been termed “mesenchymal-epithelial transition” (MET), but there are very few examples of this in the literature and most are concerned with embryogenic kidney formation [1].

The classically described process of EMT involves phenotypic change and migration of epithelial cells into the sub-epithelial mesenchyme in the lamina propria (LP) to function as extracellular-matrix producing fibroblasts/myofibroblasts [7-11]. EMT is a vital process during embryogenesis (Type I EMT), but can also be induced as a result of persistent damage and tissue inflammation [1,12,13]. There are then two subsequent outcome possibilities with active EMT: severe and even complete organ fibrosis (Type II EMT), development of a pre-malignant stroma when associated with angiogenesis (Type III EMT) [1,7-16].

This complex process of EMT involves essential changes in a wide range of proteins gained, maintained or attenuated [13], but most of the studies reported in the literature have been able to focus on only a few specific markers and are likely to be far from complete. More importantly which proteins/pathways are amendable to current therapeutics and which will require new modalities of interaction are not clearly understood. In this editorial we will focus on recent advances made in evidence of active processes of EMT in airway disease and its potential clinical importance especially in COPD, and with the use of analysis of what we know from other organ disease processes.

## Main text

We recently published that EMT is an active process in large airways of COPD patients [17,18]. Thus in smokers but especially in current smoking COPD, epithelium is in an activated state, staining strongly positive for epidermal growth factor receptor (EGFR). At the same time, the underlying reticular basement membrane (Rbm) is highly fragmented with many clefts, which itself is a structural change strongly suggestive of active EMT. Further the clefts contain cells staining for “classic” EMT markers, namely the proteolytic enzyme matrix-metalloproteinase-9 (MMP-9), mesenchymal markers including fibroblast protein S100A4 and vimentin and epithelial markers including cytokeratin [17]. EMT-marker expressing cells are also abundantly present in the basal epithelium (BE), and Rbm cells stain positively for both epithelial and mesenchymal markers [18]. Furthermore, the Rbm in large airways is hyper-vascular [14-16] i.e. give the appearance of active EMT type-III, and of course it is the large airways in COPD, where cancer formation is common, especially squamous cell carcinoma [1,6,7].

The other prime pathology associated with COPD is small airway fibrosis and obliteration, and this could potentially be related to active Type-II EMT at this site, if such would be demonstrated [8-11]. It is suggested that key players in fibrosis are fibroblasts/myofibroblasts populations. The origin of these cells is still debated in literature, if they are locally produced or bone marrow derived, but, in some circumstances EMT has been strongly implicated as a major source of these cells and can significantly change ECM characteristics of the airway wall. These changes in the ECM can alter the stiffness characteristics of the airways, perhaps paradoxically making them more floppy and vulnerable to expiratory dynamic compression and obstruction during expiration [19]. We believe that active EMT-Type-II is potentially contributing to these fibroblasts/myofibroblasts populations in the small airways hence leading to ECM changes and eventually leading to fibrosis/obliteration of these airways [8-11]. Although there are substantial reports on extracellular matrix changes in other lung diseases such as interstitial lung disease (ILD), idiopathic pulmonary fibrosis (IPF), and asthma, investigations into changes in the ECM in COPD patients are limited [19]. This warrants further studies.

Milara et al. did indeed recently report that EMT is an active process also in small airways of COPD patients and potentially contributing to small airway fibrosis [20]. In a separate study authors reported marked regression of EMT by roflumilast N-oxide, a PDE4 inhibitor in bronchial epithelial cells in vitro by restoring cellular cyclic adenosine monophosphate (cAMP) levels [21]. Wang and colleagues further elaborated this concept by demonstration of increased Urokinase-type plasminogen activator receptor (uPAR) expression, in the small airway epithelium of patients with COPD, participating in an active EMT process [22]. Authors also further demonstrated that targeted silencing of uPAR using small hairpin RNA (shRNA) inhibited cigarette smoke induced EMT in human small airway epithelial cells. However, little has yet been published as therapeutic potential to block EMT in COPD airways apart from Wang and Milara study which demonstrates so by restoring cAMP and inhibiting uPAR [21,22].

We recently reported [23] in a randomized controlled trial that inhaled corticosteroid fluticasone propionate given over six months suppressed EMT-related changes in large airways of COPD patients. This trial showed marked reduction in Rbm fragmentation (core structural hallmark of EMT), epidermal growth factor receptor (EGFR), basal epithelial cell S100A4 expression and in Rbm cell S100A4 and MMP-9 in the active inhaled corticosteroids (ICS) compared to placebo [23]. This is the first study reporting anti-EMT effects of inhaled corticosteroids in COPD, where of course they are widely used as putative “anti-inflammatory” agent, although that mode of operation is far from convincing [24]. In human epidemiological studies it is strongly suggested that patients on inhaled corticosteroids, albeit only at high doses (as used in our study), are associated with an appreciable (50%) reduction in the risk of lung cancer [25-27]. We suggest EMT might be the process by which this effect of ICS occurs. If this is true, it has huge implications for therapeutic and public health policy, since it is strongly suggested in literature that patients on ICS are associated with a decreased risk for lung cancer. It is suggested that statins may also have similar effects on EMT in COPD, since lung cancer risk decreases in COPD patients who are on statins [28], this warrants further studies.

We are not alone in recognising such possibilities. Thus, recently in lung adenocarcinoma, vaccination against drivers of EMT was proposed as an immunotherapeutic approach against tumor progression, which is also attracting interest from pharmaceutical industries and in fact adenocarcinoma is now getting more common compared to squamous cell carcinoma. The investigators developed an immunotherapeutic approach to target a major driver of EMT, the T-box transcription factor T (also known as brachyury) [29]. This therapeutic phenomenon is currently being tested in patients with advanced carcinomas in the context of a Phase I clinical trial [30,31]. This approach is expected to generate an effective T-cell (CD8+) response that selectively eradicates tumor cells expressing the EMT driver of choice and undergoing EMT switch. When employed in combination with conventional chemotherapy eliminate epithelial tumor cells [29,30]. It is of interest and relevance in this context that over 90% of human cancer arises in epithelia (eg breast, colon, stomach, liver, prostate, ovary/fallopian tube, bladder), and the involvement of EMT in all of these may be a central paradigm [32-34]. COPD-related cancer may well be just another example of this core principle of unstable epithelium in the context of tissue inflammation and/or chronic stimulation [19].

In another study epigenetic based inhibition of EMT by sorafenib has been reported in human lung epithelial cells (A549, a lung adenocarcinoma cell line). Sorafenib (Nexavar or BAY 43-9006) is a small molecular inhibitor that targets a number of serine/threonine and receptor tyrosine kinases, and is the first oral agent approved for the clinical treatment of a variety of tumor types since 2005 by the US Food and Drug Administration. In this study, the authors reported that sorafenib inhibited TGF-β1-induced EMT by increasing histone acetyltranferase (HAT) expression and by decreasing histone deacetylase expression possibly via inhibition of Ras/Raf MAPK and ErbB signaling [35]. Camera et al. also suggested TGF-β1/Smad2/3 as a potential therapeutic target for EMT [36]. Yang et al. further demonstrated that crosstalk between muscarinic acetylcholine receptor (mAChR) and TGF-β1 can lead to EMT induction in lung epithelial cells (A549) suggesting a role of non-neuronal cholinergic system in EMT and a potential novel therapeutic target for EMT [37].

Byers and colleagues reported that in non-small cell lung cancer (NSCLC) inhibition of Axl-RTK (another receptor tyrosine kinase) by SGI-7079 lead to decrease in growth of NSCLC tumors. The authors suggested Axl-RTK as a potential therapeutic target for overcoming EGFR inhibitor resistance associated with mesenchymal transition [38]. Similar effects of sorafenib are observed in liver [39] and urothelial carcinoma *in situ* [40] by targeting STAT3 and urokinase plasminogen activator (uPA). Recently in idiopathic pulmonary fibrosis, efficacy of Nintedanib (multiple tyrosine kinases inhibitor) and pirfenidone (an anti-fibrotic and anti-inflammatory drug) has been reported in improving lung function [41,42]. It is quite possible that these drugs might also affect the process of EMT or are inducing their therapeutic effect at least partly by blocking EMT, since EMT has been shown to be active in IPF [43]. Nintedanib works mainly by inhibiting angiogenesis so it may have implications for EMT-Type-III where angiogenesis is prominent and on the other hand Pirfenidone is more anti-fibrotic in action so may have implications for EMT-Type-II. These warrant further studies, as they may have both anti-fibrotic and anti-cancer effects through EMT.

## Conclusions

EMT is an important process in airway disease especially in smoking related COPD, where it might be contributing to small airway fibrosis and large airway epithelial cancers. However there are very few studies reporting anti-EMT effects of different drugs and data are especially sparse from *in vivo* human clinical investigations. Most of the conclusions are drawn from *in vitro* studies. Therapeutic approaches to EMT are still in their infancy and we don’t know which protein or pathways we should target to block this profound epithelial plasticity. Given the fact that disease-associated EMT is of two types, it becomes more complicated to tease out what proteins/pathways are contributing independently to fibrosis and cancer and what therapeutic modalities will they require since BOTH types share the EMT proteome, but empirically seem to differ only in the neo-angiogenesis component.
